# COVID-19 in the United States as affective frame

**DOI:** 10.3389/fpsyg.2022.897215

**Published:** 2022-08-09

**Authors:** John Protevi

**Affiliations:** ^1^Department of French Studies, Louisiana State University, Baton Rouge, LA, United States; ^2^Department of Philosophy, Louisiana State University, Baton Rouge, LA, United States

**Keywords:** affect, enaction, Deleuze, Foucault, biopower, racism, COVID-19, critical phenomenology

## Abstract

In this paper I attempt to contribute to the developing field of “political philosophy of mind.” To render concrete the notion of “affective frame,” a social situation which pre-selects for salience and valence of environmental factors relative to a subject’s life, I conduct a case study of a deleterious socially instituted affective frame, which, during the early days of the COVID-19 pandemic in the United States, produced individuated circumstances that came crashing down on “essential workers” who were forced into a double bind. We saw here an untenable and ultimately fatal situation that forced a choice between, on the one hand, increasing the risk of their failing to provide financial support for their family if they quit their job or reduced their hours, and on the other, increasing their risk of contracting the virus by continuing to work. The case study will thus be itself an affective frame that will bring to the fore for its readers a nexus of harmful social practices of contemporary American society. Form is reinforced by content here, as this particular affective frame brings forth a further emphasis on affect when we focus on workers simultaneously socialized into roles as breadwinners and as members of the caring professions. For those people, quitting work becomes even more difficult as they come to affirm their self-identity of being providers of affective labor for those in their care at work and of being the affective anchor of family life at home, the one who financially helps keep a roof over the heads of their loved ones as well as being the emotional backbone of the family. Hence the affective frame of “essential workers in Covid times” renders salient and affirmatively valenced their affectively laden self-image as caring helpers of those in need, at home and at work.

## Introduction

In May 2020, as COVID-19 swept through southern Louisiana, I read an article in my local newspaper that was both heart-breakingly concrete and illustrative of wider trends (De Robertis, 2020). The article relayed the story of the life and death of Ms. Shenetta White-Ballard, a nurse at a senior care facility and hence an “essential worker,” who died after exposure to COVID-19 at work. The powerful affective tenor of the reporting immediately pulled me in. Ms. White-Ballard was described by friends and family as “terrified” of the risks of contracting Covid at work, “responsible” for her family’s financial viability, and as having a “beautiful heart” full of concern for her family and her patients. I would like this article to serve as a tribute to her courage as well as a critical account of the social forces that shaped the affective frame within which she enacted her agency.

## Toward a political philosophy of mind

The philosophical framework of this piece the enactive wing of the nascent movement to establish a “political philosophy of mind.” A political philosophy of mind integrates third-person political analysis of subjectification practices with the traditional philosophy of mind topics of third-person scientific investigation of sub-personal neural, endocrinological, and somatic mechanisms, first-person phenomenological explorations of experience, and studies of second-person interaction, as in “participatory sense-making” (De Jaegher and Di Paolo, 2007). A successful political philosophy of mind must avoid reduction (collapsing the sub-personal and first-person perspectives), individualism (society is just an aggregate of individual subjects), and strict structuralism (subjective actions and experiences are mere consequences of one’s social position).

The move toward a political philosophy of mind within the enactive framework can be said to have begun with Shaun Gallagher’s discussion of the “socially extended mind.” Gallagher (2013) looks at “enactive processes (e.g., social affordances)” which, for him, go beyond second-person relationships to consider “institutional structures, norms, and practices.” Taking a critical turn, Gallagher asks us to “look more closely and critically at how social and cultural practices productively extend or, in some cases, limit mental processes.” In his 2020 book, *Action and Interaction*, Gallagher cites Iris Marion Young on oppression and Charles Mills’ critique of ideal theory to support his call to “give cognitive science a critical twist.”

A second important figure is Jan Slaby, who moves from his work on critical neuroscience to the political philosophy of mind. Slaby and Gallagher take up the notion of institutional extension in a 2015 article, in which they state, “The rational human subject is not an exclusively biological entity-it is an entity coupled with other biological individuals and with various institutions, tools, procedures, and practices that enable cognition (Slaby and Gallagher, 2015).” The political edge comes when we realize that such an extension is not always enabling; in the article in which he coined the term “political philosophy of mind,” Slaby (2016) points out that many social embeddings produce affective reactions that inhibit rather than develop human potentials:

We can… distinguish enabling from disabling social structures, we can assess the extent to which social domains work to establish mental patterns that, in the long run, are enabling, conducive to individual and collective flourishing, or whether they instead create unhealthy dependencies, bind us to oppressive routines, maintain inequality, destroy community ties, or lead to emotional and mental habits that are harmful to us or our loved ones.

In *The Mind-Body Politic* (2019), Michelle Maiese and Robert Hanna develop further the move that calls for a political philosophy of mind. Maiese and Hanna have a deep embodiment thesis [we are “essentially embodied minds,” they write at Maiese and Hanna (2019, p. 2); at 41 they speak of “minded human animals”] in which social and material encounters shape the minds and bodies of humans, so their first-person experience and second-person interaction, while not *determined* politically, are nonetheless politically conditioned as our inherent neural and somatic plasticity is molded by our cultural niches.

In pursuing a political philosophy of mind, Maiese and Hanna criticize overly cognitive notions of ideology (e.g., the belief-centered work of Tommie Shelby) and call for the inclusion of affect and emotion in considering the frames of reference that condition our engagement with the world (Maiese and Hanna, 2019, p. 260ff; for a call for “affective ideology” see Protevi, 2016). Hence, instead of belief-centered ideology, they propose “affective frames” as a more effective way of analyzing the experiences and interactions of deeply embodied and socialized minds. In other words, for Maiese and Hanna, affect in its aspects of salience (importance relative to the background) and valence (impulsion to approach or avoid), is the key to understanding how essentially embodied minds operate in their physical and social worlds.

Drawing on the work of Slaby (2016), Maiese and Hanna describe affective frames as “spontaneous, non-inferential, and pre-reflective way of discriminating, filtering, and selecting information” (Maiese and Hanna, 2019, p. 41). This filtering is accomplished in terms of the salience or importance of the environmental element for an organism’s value systems: “Affect draws our attention to specific features of our surroundings and implies a ‘dynamic gestalt or figure-ground structure’ whereby ‘some objects emerge into affective prominence, while others become unnoticeable”’ (Maiese and Hanna, 2019, p. 41; internal quotations from Thompson, 2007, p. 374).

Continuing with an enactivist approach, Maiese and Hanna insist that affective framing is distributed rather than brain-centered: “Affective framing is best understood as distributed over a complex network of brain and bodily processes … [including] metabolic systems, endocrine system, the musculoskeletal system, and the cardiovascular system” (Maiese and Hanna, 2019, p. 41–42). Such a distributed somatic system is in essential interaction with the environment, which for humans very often has crucial social dimensions. Hence, an affective frame can be instantiated in a social institution that makes some affective stances more likely: “A social institution thereby significantly modulates affective framings, substantively molds overall bodily comportment, and literally shapes the minded bodily habits of the subjects involved” (Maiese and Hanna, 2019, p. 56).

As we can see, an affective frame is doubly distributed, spread across the social institutions and bodily systems that make it up. The episodes of our lives, then, are individuations of those distributed systems, which, unfortunately, may become out of synch with our wellbeing. Thus, one can all too often find “autonomous, self-sustaining structures of affective framing or habit that are actually *in essential conflict* with basic values associated with fundamental human needs and overall wellbeing” (Maiese and Hanna, 2019, p. 59; italics in original).

## Methodology and ethics of case studies

In this paper I will conduct a case study of a deleterious socially instituted affective frame, which, during the early days of the COVID-19 pandemic in the United States, produced individuated circumstances that came crashing down on “essential workers” who were forced into a double bind, a no-win situation with an unavoidable entanglement of financial and health risks. We saw here an untenable and ultimately fatal situation that forced a choice between, on the one hand, increasing the risk of their failing to provide financial support for their family if they quit their job or reduced their hours, and on the other, increasing their risk of contracting the virus by continuing to work.

The case study will thus be itself an affective frame that will bring to the fore for its readers a nexus of harmful social practices of contemporary American society. Form is reinforced by content here, as this particular affective frame brings forth a further emphasis on affect when we focus on workers simultaneously socialized into roles as breadwinners and as members of the caring professions. For those people, quitting work becomes even more difficult as they come to affirm their self-identity of being providers of affective labor for those in their care at work and of being the affective anchor of family life at home, the one who financially helps keep a roof over the heads of their loved ones as well as being the emotional backbone of the family. Hence the affective frame in which “essential workers in Covid times” found themselves renders salient and affirmatively valenced to them their selves as caring helpers of those in need, at home and at work.

In my view, case studies have epistemological benefits: they render concrete what is otherwise a study of patterns rather than an investigation of a particular event. This in turn implicates an ontological point about the way concrete lives are lived out as the nexus of processes whose patterns can be treated statistically, but which are concretized as a series of events one experiences (Protevi, 2009 contains several case studies).

Alongside its epistemological benefits, a case study raises ethical concerns of privacy and social positioning of authors and their subject. I don’t think there’s precisely a privacy concern here, as the case study relies on a published newspaper article; the people quoted therein presumably consented to have their words used and their names cited. However, there is a change from reporting a set of facts—Ms. White-Ballard’s death and the comments made about it by the people quoted—to using the story of her death to render concrete the dilemma of “essential workers.” I could anonymize the article by cutting out the case study entirely to just talk about the structural problems of juggling financial and viral exposure risks. But that would stay on the level of statistical patterns which loses the emotional connection to a life cut short by the way those processes, whose patterns can be discussed statistically, came crashing down on a singular life, that of Ms. White-Ballard.

Regarding social positioning, I’m conscious of being a White scholar writing on a Black subject. In treating the testimony surrounding Ms. White-Ballard’s death, am I making a spectacle of black suffering? Lindsey Stewart’s challenging new book, *The Politics of Black Joy* (Stewart, 2021), calls attention to a “neo-abolitionist” focus on Black pain to the exclusion of Black joy. I don’t believe we should take Stewart’s challenge to mean that any focus on social structures that disproportionately harm Black people is to be avoided; in other words, “neo-abolitionism” is not off the table entirely. Rather, its focus on suffering should never be allowed to overwhelm our reverence for the entirety of the lives one discusses; in Ms. White-Ballard’s case, the fullness of her humanity shows through in the beautiful tributes conveyed by her friends and family.

## Forecast of the article

I will use an outside-in method so we can see the full dimensions of the situation in which COVID-19 served as an affective frame for Ms. White-Ballard’s death. I begin with a sketch of the Deleuzean metaphysical background for individuation in distributed systems, and then move to a closer look at affective frames and embodied minds. From there I will provide a Foucauldian analysis of the long development of “biopower” regimes for public health and disease management, culminating in the development in the last 40 years of a regime of neoliberalism, financialization of daily life, and risk management as we are subjectified as “self-entrepreneurs” responsible for health decisions for self and family.

Moving still closer to the present, I will look at race and COVID-19 in the United States, specifically the notion of “pre-existing conditions” or “co-morbidities” and their relation to “weathering,” the thesis that chronic stress in populations suffering anti-black racism will accelerate aging as measured by telomere length. Hence, when faced with the COVID-19 pandemic, those who are negatively racialized and precariously employed, deprived of a robust social safety net so that they are left as life-management agents responsible for both self and family, face an intense entanglement of financial and viral risk management. Turning briefly to enactive accounts, we see that searching for epistemic solutions itself imposes a physiological cost that contributes to weathering.

Finally, I will show how all these factors crystalize in the case of Shenetta White-Ballard, who, having strongly identified with the affective components of her roles as providing necessary care to her family and her patients, and who, when faced with the conflicting risks of family financial ruin by stopping work and contracting the virus by continuing to work, chose the latter, and ultimately died of COVID-19 in May 2020.

## Deleuzean metaphysics of individuation in distributed systems

Case studies are an under-used tool in philosophy, as opposed to thought experiments such as brain transplants, brains-in-a-vat, zombies, and others. Case studies do not aim at identifying the necessary and sufficient conditions for an essential distinction, as do thought experiments. Instead, case studies reveal the outlines of concrete problems, which are the points of intersection of “multiplicities,” a Deleuzean term of art which means a “problematic” field in which linked rates of change create conflicting pressures, so that any one move changes the conditions for future moves and no one solution exhausts the potentials for future creatively different solutions. In other words, Deleuzean problems, the problems of life, cannot be “solved” once and for all; they can only be dealt with (I present several case studies and develop their philosophical background at some length in Protevi, 2009, 2013).

A key Deleuzean concept in thinking how case studies investigate the relation of problems and solutions is “individuation,” which needs to be thought of as a process of transient emergence via the integration of a dynamic differential field. You don’t determine individuality by looking at already formed substances and placing them in a categorial system; you look at the process by which an individual emerges. A simple physical image of individuation used by Simondon (2020) is crystallization of a super-saturated solution. A crystal is formed by bringing together, by integrating, the potentials of the ever-changing meta-stable field that is the super-saturated solution. But these potentials are not there in already individuated form; they are the potentials embedded in difference gradients. There is literally nothing, no-thing, there prior to the crystal, that is, nothing crystalline.

Deleuze has a threefold formula to express this ontology of individuation from distributed systems: beneath an *actual* substance we find *intensive* “impersonal individuations” and beneath them we find *virtual* “pre-individual singularities.” *Virtual* fields are composed of differential elements, differential relations, and singularities: networks of linked rates of change with thresholds or turning points. The virtual doesn’t exist, but provides the “diagram” for individuation processes, which are the only things that do exist. Virtual diagrams stay in reserve; no one solution exhausts their potential for future creative solutions. *Intensive* individuation processes are flows of matter and energy, driven by differences or gradients, which produce individuals as transient emergences. In other words, individuation is the process by which a system self-organizes and exerts a “focus” as it constrains its components. *Actual* substances are systems at equilibrium or locked into habitual patterns. They are the cooled off or mature product of intensive individuation processes: think of rocks congealing from lava flows, or mature differentiated cell types having developed out of earlier totipotent stem cells, or indeed, the mature habits of a person set in his or her ways as the loss of earlier flexibility. And these habits are not just behavioral; they are perceptual as well: you can lose the ability to do anything more than “recognize” in a situation the things that fit into your pre-conceived categories; you can lose the capacity to feel what might be newly possible. Such choices though are always socially constrained; indeed, our case study is of a double bind in which all choices are bad one.

## Affective frames and bodies politic

Our next step in providing the theoretical context for our case study moves from a general metaphysical scheme to the incarnation in bodies of habits formed via social practices. As we have noted, Maiese and Hanna develop further the enactivist move broached by Gallagher and Slaby, from a focus on perceptual-motor linkages in individual organisms to second person participatory sense-making to third person political philosophy of mind. In previous work, I have used the term “political affect” to denote ways “in which a body is patterned by the social system into which it is acculturated” (Protevi, 2009, p. 32; quoted at Maiese and Hanna, 2019, p. 43).

I’d like to take some time here to lay out some thoughts on political affect, condensing what I did in Protevi (2009), which has a full bibliography of relevant background work. Affect is both openness and feeling, being affected. Affect is the feeling for variation; it is the intensive as opening access to the virtual, to the differential field or multiplicity of the situation. The intuitions generated here are the integration of the differential situation. This will prepare us to see the contours of the emotional experience undergone by Ms. White-Ballard, (1978) who was described as “terrified” of the risks of contracting Covid at work, “responsible” for her family’s financial viability, and as having a “beautiful heart” full of concern for her family and her patients.

In a political affect perspective, humans aren’t substances with properties, but singular patterns of social and somatic interaction. The embodied and the embedded aspects of our being intersect—we are bodies whose capacities form in social interaction. That is, our biology, our nature, is to be so open to our nurture that it becomes second nature—that’s the upshot of the intersection of plasticity and niche-construction. And it’s in this intersection of the social and the somatic that subjectivity and selfhood emerge. In much simpler terms, “singular patterns of social and somatic interaction” means that we are what we can do with others—the way our embodied capacities, which develop in the history of the social interactions we have had up to the present, intersect with the similarly constituted embodied capacities of the others we now encounter and the social affordances of the environment. The complexity and creative potential of these encounters is such that we don’t know what we are until we experiment with what we can do. This emphasis on open-ended, creative, and unpredictable experimentation is part of the meaning of the at first glance very strange Deleuzean term, “transcendental empiricism”: you have to explore the virtual realm by reflecting on progressively accomplished actualizations.

My analyses so far in this paper have been abstract. In other works, I have always insisted that when cognitive science looks at the extended and embedded mind, it needs to have a political analysis of the subjectification processes in a population (Protevi, 2009, 2013). Without a population variation perspective, we risk relegating the cultural to a storehouse of heuristic aids for an abstract problem-solver who just happens to be able to interact successfully with the people and cultural resources to which it just happens to have access. So, we need to analyze not simply technical training for cognitive capacities in a restricted sense, but also the training necessary for acquiring positive and empowering emotional patterns, thresholds, and triggers. Hence, we need to think in terms of a range of subjectification practices that are distributed in a society at various sites (family, school, church, media, playground, sports field, and so on) with variable goals, intensities, and efficacies. These multiply situated practices resonate or clash with each other and with myriad other practices (gendering, racializing, and so on). But even this is still too simple, as these subjectification practices also enter complex feedback relations with the singular body makeup (genetic and epigenetic) of the people involved. All the way down, we are biological and social; we are “bodies politic.”

To keep contact with the experiential dimension as we develop our political philosophy of mind, we should turn to “critical phenomenology” (among the classics are Fanon, 2008; de Beauvoir, 2012; for further developments see Ahmed, 2007; Weiss et al., 2019; Guenther, 2019). Noting that classical phenomenological analyses of embodiment, temporality, intentionality, perception, intersubjectivity and historicity by Husserl and Merleau-Ponty, echoed in Noë, 2005 and other enactivists, presuppose a neutral or unmarked, socially unsituated, body-subject, critical phenomenology loops us back to the first-person experience of individuals subject to politically analyzable socially denigrated subjectification practices. Critical phenomenology shows us the necessity of a move from the unmarked abstract subject to a differential field of embodying practices being actualized in a differentiated field of concrete embodied subjects. Reflecting on the early days of phenomenology, critical thinkers show that the unmarked subject had a hidden content of white, male, able-bodied, and economically secure subjects moving in a world fitted to their actions such that whiteness, maleness, able-bodiedness, and economic security was rendered invisible. It’s only in tracing the movement from Sartre’s gaze of the Other to Beauvoir’s male gaze to Fanon’s white gaze and other such phenomena of “sociogeny” (Fanon, 2008, p. xv) that we see racializing, gendering, “able-ing,” and securitizing as practices producing whiteness as well as blackness, masculinity as well as femininity, able-bodiedness as well as disability, and security as well as precarity.

## Biopower

Previously well-known among academics, Michel Foucault’s term “biopower” achieved new public prominence in the Covid pandemic as commenters reacted to governments enacting public health measures beyond those already in place, which had become with the passage of time naturalized or invisible. For Foucault, the terms biopower and biopolitics referred to long historical modifications of regimes of power-knowledge, or roughly speaking, theoretically guided practice. In his middle period works of 1975-78—*Discipline and Punish, “Society Must Be Defended,” History of Sexuality, vol. 1*, and *Security, Territory, Population*—Foucault distinguished several overlapping and intersecting forms of power-knowledge; any one historical era institutionalizes a different blend of these forms, rather than simply replacing one with another.

When operating in a sovereign power modality, governments deduct taxes and compel obedience of law by force; the slogan of sovereign power is “take life or let live.” Disciplinary power operates in institutional settings and aims to increase productive obedience by individualizing examination, the establishment of norms, and exercises to reduce deviation from those norms (Foucault, 1979). Sexuality is a form of power-knowledge that, rather than operate by sovereign command or by disciplinary asymmetrical orders, instead induces people into acts of reflective self-subjectification, asking them to find their truth in their sexual desires. Foucault (1978) locates sexuality at the intersection of individualized discipline and population-level biopower, our final concern. Biopower regimes manage the biological realities of a population in its environment; the slogan here inverts that of sovereignty, whose “take life or let life” is transformed into biopower’s “foster life or let die” (Cisney and Morar, 2016).

While a transitive, externally directed, and adversarial “war” frame for power fit the transition from sovereign power to disciplinary power in *Discipline and Punish*, power relations became more subtle in *History of Sexuality, volume 1*, as instead of highly constrained transitive commands of discipline (“take this test, perform these exercises”) the practices making up the sexuality framework (*dispositif*) very often “induced” a self-subjectification in which subjects are constituted in and by the search for personal truth in sexual desires. At this time, then, Foucault introduced “governmentality” as a frame for reading intransitive, non-adversarial, self-subjectifing power relations (Lemke, 2019). In governmentality situations, third-person-directed and second-person-instantiated practices condition the way in which I relate to myself in first-person lived experience. Governmentality is defined as “conduct of conduct,” that is, leading people to govern themselves, to self-subjectify in a particular way. Foucault’s genealogy of governmentality in *Security, Territory, Population*, Foucault (2007) and *Birth of Biopower* (2008) disentangled several strands: religious self-subjectification as led by “pastoral power”; sexual self-subjectification as an agent searching for truth in sexual desires; liberal self-subjectification as an agent in pursuit of self-interest; and finally, neoliberal self-subjectification as a “self-entrepreneur” (Lemm and Vatter, 2014).

It will be germane to our study, then, to see the way in which a further concretion of the practice of being a self-entrepreneur comes from the inducement to self-subjectify as the agent of your and your family’s health choices by being an active partner with health care providers. A further twist then comes when your financial situation depends on and is entwined with your work in the health care wing of “the caring professions.” Here we allude to a self-subjectifying practice, unmentioned by Foucault, but later developed by theoreticians of “emotional labor,” in which some are led to identify with the caring practices they perform at work, as if one were to say, “this job comes easily to me, as I am a caring person at heart” (Hochschild, 1983; Ashforth and Humphrey, 1993; Johnson, 2015, p. 113, explicating Hochschild, warns of “the mismeasurement of emotional labor as a natural disposition with its own intrinsic rewards”). A full development of this notion, beyond what we can do here, would entail investigating the complex relations of paid emotional labor with traditional feminization practices that thematize caring as essentially or at least characteristically female; Gilligan (1982) would be a key reference here.

## Risk, financialization, and neoliberalism

The core of my analysis is that contemporary workers deemed “essential” during COVID-19 and hence unable to work from home, were caught in a double bind of navigating increased risk of viral exposure at work to mitigate their exposure to financial risk incurred by taking on debt in a world of stagnating wages. Here I will briefly treat the intertwining of risk and financialization in the neoliberal system.

Risk has been a popular social theory topic for the past 40 years or so; Jacob Hacker’s *The Great Risk Shift* (2006) is a noteworthy treatment of the destruction of the social safety net focused on the Fordist bargain and its “family wage.” Melinda Cooper’s (2019) *Family Values* refuses any left nostalgia for Fordism, and instead provides detailed case studies of the way in which, during the neoliberal restructuring of society, “instead of trying to revive the family form of the New Deal, they [neoliberal policy makers] tried to revive the much older poor law tradition of family responsibility, which identified marital and kinship relations as the proper source of economic security and a suitable alternative to the welfare state” (Cooper and Mable, 2018). Debt, risk, and Covid all come together, then, in an affective framing that pushed “essential workers” to accept increased Covid risk exposure by continuing to work due to their need to service financial debt.

The major sociological work on risk is Ulrich Beck (1992) *Risk Society*. For Beck, modern society is faced with managing risks that the very operation of the system has created. Mitchell Dean (1998), from a Foucauldian perspective, demurs. For Dean, rather than being system-wide, risk is best thought of as managed in rational calculation in small assemblages. Curran (2013a,b, 2016), for his part, criticizes Beck’s lack of class analysis. Curran calls for “critical risk analyses” such that “differentials in economic power constitute a key form of social power for avoiding certain risk positions and rendering others exposed to the worst of the emerging damages” (Curran, 2013b, p. 75). Beyond Curran’s work, we also see further research looking at risk in intersectional terms, adding race and gender to class (Luft, 2016; Olofsson et al., 2016; cited by Zinn, 2018).

“Financialization” refers to the way cost-benefit analyses saturate society at all levels, from individual to household to city, state, federal, and international (Martin, 2002 is an early classic on individual and household level financialization). A recent noteworthy analysis to the literature on debt, risk, and financialization is Adkins (2018) who shows that credit is no longer extended with a view to full repayment of the debt. Rather, creditors seek monthly revenue streams which can be securitized and sold on various secondary markets. Faced with declining purchasing power, workers now commonly leverage salary to take on the debt (credit cards, car loans, and mortgages) that allows them to make ends meet. You don’t work hand-to-mouth anymore; you work hand-to-check-to-credit-application. Cooper and Mable (2018) comments on these developments in which neoliberal policies produce private debt:

We can also observe multiple ways in which cuts to public funding in healthcare, education, and welfare have pushed people back toward kinship-based forms of self-care and mutual support and how the expansion of consumer credit has turned household deficit-spending into a substitute for state deficit-spending. Today, family responsibility very often takes the form of intergenerational debt where parents and other family members are actively enrolled in the debt obligations of children, signed up as guarantors or required to post their housing wealth as collateral to fund the social mobility (or simply stasis) of younger generations.

“Neoliberalism” is sometimes said to have degenerated into an empty epithet, just a term for critics of contemporary capitalism to use to sound sophisticated (see Mirowski, 2018 for a superb rebuttal to those claims). For purposes of this article, I follow Foucault’s analysis, laid out in *The Birth of Biopolitics* (2008). Foucault writes that neoliberals reject the classical liberal notion that humans have a natural propensity to truck and barter, such that governments should step back to allow this natural process to take place. They rather assume, Foucault writes, that competition is fragile and needs state intervention to set up its conditions.

Contra Maiese and Hanna (2019, p. 103), hence, I don’t think that neoliberalism is Hobbesian individualism (i.e., a social contract based on the need for government to allow personal security and economic collaboration via securing stock and enforcing contracts that people naturally desire). Rather, neoliberalism does not rest on the solidity of individualism, but on its fragility: a social fabric linking individuals is always on the brink of collapsing into collectivism. Hence neoliberalism is the inverse of Hobbes; for the neoliberals, cooperation is fragile and needs state oversight to guarantee that it does not break down.

Furthermore, the classical political economy standpoint in which labor power is a commodity for purchase (e.g., Harvey, 2007, p. 20) cannot be reconciled with Foucault’s treatment of Gary Becker’s human capital theory. Becker undercuts the (Marxist) treatment of commodified labor power by treating individuals as themselves firms. Salaries become returns on investment in skills; education becomes development of “human capital.” Picking up on Becker enables Foucault to develop the notion of individuals as “self-entrepreneurs,” for whom each action is either investment or return on human capital. In this way, Foucault can inscribe neoliberal governmentality in his history of subjectification practices. In other words, for Foucault, neoliberal governmentality conducts our conduct by inducing us to subjectify ourselves as self-entrepreneurs concerned with obtaining a return on our human capital (Foucault, 2008, p. 221–226).

## Racial “weathering”

Governmentality practices that induce self-subjectification as a self-(health)-entrepreneur would start with contemporary American mandates for individual health insurance packages purchased on government subsidized insurance markets but extend to such matters as legalizing direct-to-consumer medical advertising (“ask your doctor if X is good for you!”), state support of medical research, laws and regulations for reproductive health, and on and on. Insofar as producing the conditions for self-entrepreneurship allows for management of an individualized population, American biopower practices depend on analyzing rates in a vast multiplicity of myriad dimensions. Just to begin, multiple agencies at local, state, and federal levels analyze the way in which citizens with such and such a demographic profile (income, occupation, residence, gender, race …) consume such and such a level of health care (number of office visits, rate of prescription refills …) and have such and such health outcomes (sickness leading to days missed at work, life expectancy, cost of end-of-life care).

Insofar as the American biopower multiplicity includes dimensions affected by political practices of racialization, gendering, and other forms of “deep embodiment,” we here see a biopolitics of “differential vulnerability” (Lorenzini, 2020). Recall that the slogan of biopower is “foster life and let die.” Lorenzini shows that Foucault’s position in *“Society Must Be Defended”* (Foucault, 2003) amounts to racism as “a way of introducing a break into the domain of life taken over by power: the break between what must live and what must die.” In biopolitics, racism “fragments the biological continuum” (we all are living beings with biological needs) to create hierarchies between different human groups, and thus differences in the way in which some are exposed to increased risk of death.

Here we see biopower as “letting die” as opposed to sovereign execution; however, see Mbembe, 2019 on contemporary sovereignty and “necropolitics,” the construction of geographical and social zones where racialized bodies are marked not just for withdrawal of biopower support, but also for what we could call easy transitive death operations. Hence, we mustn’t think biopower is the only form of governmental action on biological processes going on today. Nonetheless, following Lorenzini, we can say that the differential exposure of human beings to health and social risks is, per Foucault, a salient feature of biopolitical governmentality (see Weheliye, 2014 for criticism of Foucault’s use of the concept of racism).

The “deep embodiment” biological effects of the racializing subjectification practices analyzed by critical phenomenology links racial discrimination to “weathering” or premature aging, which must be considered in discussing Covid as affective frame. Fanon had already talked about the impingement of body image on body schema (Fanon, 2008, p. 90–91). With weathering, we find an instance of “political physiology” that fits with the mind-shaping and body-shaping theses of Maiese and Hanna (2019), Chowkwanyun and Reed (2020) is already a classic in its insistence on warning against simple ascription of Covid vulnerability to race without adequate context: “disparity figures without explanatory context can perpetuate harmful myths and misunderstandings that actually undermine the goal of eliminating health inequities.”

Chowkwanyun and Reed cite three main dangers to sheer racial ascriptions. First, we risk ascribing inherent biological differences to what is a bio-social event (the saying that “there is no such thing as a natural disaster” was made popular during Hurricane Katrina; the same thing holds for Covid). Similar warnings hold against ascribing Covid racial discrepancies to behavior or to sheer residential patterns. The latter risks the psychological phenomenon of “place-based stigma,” when in fact we should be looking at “place-based risk,” that is, material factors responsible for poor health outcomes in the location [For example, although Chowkwanyun and Reed do not go into this level of detail, I believe they would be sympathetic to my pointing out that neighborhoods with high Black population are not “bad neighborhoods” as if the mere presence of Black people hurts their reputation; some of them are literally poisoned by air pollution contributing to high respiratory disease rates and related high Covid rates (Wu et al., 2020, cited in Olumhense, 2020)].

To fight this, Chowkwanyun and Reed insist, we need socioeconomic status data to contextualize disease rates. “Complementary SES information will clarify how racial and class forces are intertwined—and when they are not—in the case of COVID-19. In general, members of minority populations are disproportionately likely to have low SES and are likely to have the most undesirable health outcomes.”

Most interestingly for us, however, the authors now turn to the concept of “weathering” for cases when SES does *not* explain COVID-19 racial disparities:

One possible explanation is the role of stress and what public health researcher Arline Geronimus has termed “weathering,” or advanced aging caused by bodily wear and tear from fight-or-flight responses to external stressors, especially racial discrimination (internal citation to Geronimus et al., 2006). Weathering has been linked, in turn, to cardiovascular disease and diabetes, two conditions that have been associated, in preliminary research, with elevated risk for severe COVID-19 (Chowkwanyun and Reed, 2020).

Chowkwanyun and Reed conclude, “In sum, to mitigate myths of racial biology, behavioral explanations predicated on racial stereotypes, and territorial stigmatization, COVID-19 disparities should be situated in the context of material resource deprivation caused by low SES, chronic stress brought on by racial discrimination, or place-based risk.”

Let us now turn to the concept of “weathering” laid out by Geronimus in numerous publications. First, let us note that weathering is measured by telomere length. Telomeres are repetitive sequences of non-coding DNA that, during cell division, protect coding DNA on the chromosome. However, each time a cell divides, the telomeres become shorter so that, eventually, the telomeres become so short that the cell can no longer divide. Geronimus et al. (2010) studied US Black women and telomere shortening; note the emphasis on “perceived stress.”

We hypothesize that black women experience accelerated biological aging in response to repeated or prolonged adaptation to subjective and objective stressors… We also perform a first population-based test of its plausibility, focusing on telomere length, a biomeasure of aging that may be shortened by stressors. Analyzing data from the Study of Women’s Health Across the Nation (SWAN), we estimate that at ages 49–55, black women are 7.5 years biologically “older” than white women. Indicators of perceived stress and poverty account for 27% of this difference.

Following up on her early research, Geronimus (2013) calls for a “deep integration” approach to public health, linking epigenetics to weathering, for it’s not just intra-uterine or very early childhood that is corporeally inscribed. Rather, lifelong chronic stress can result in an accumulated “allostatic load”; the concept of weathering then claims that racial discrimination is a cause of heightened allostatic load compared to other segments of population.

## Predictive processing and chronic stress

With the notion of allostatic load, we come across an interesting connection with the currently prominent cognitive science school of “predictive processing.” Predictive processing says the brain seeks to minimize the gap between predictions of the environment and arriving sensory information that serves as error correction to those predictions. Current work looks at exteroceptive (from environment), proprioceptive (from musculoskeletal sensations), and interoceptive (internal milieu) prediction and sensation (Seth and Friston, 2016). Error correction occurs as perceptual inference or active inference. Perceptual inference is changing beliefs to fit incoming sensation, thus reducing prediction errors from the previous cycle. Active inference is action to change sensation to fit beliefs. This could take the form of changing head position to hear or see better, or it could be dynamic anticipatory control of interoception. In unpredictable and dangerous environments, we should note, such error correction carries a heavy physiological cost resulting in allostatic load.

Peters et al. (2017) is a very thought-provoking article that examines such physiological costs. The authors examine the “mathematical, neurobiological, and medical aspects of uncertainty.” In other words, they bring together the Bayesian Brain, the Selfish Brain, and what we could call the Stressed Body. The Bayesian Brain concept looks at how brains minimize “free energy” in its information theory aspect as uncertainty in strategy choice for perceptual or active inference that would reduce prediction errors, while the Selfish Brain concept looks at the physiology of the stress response that provides cerebral energy for via increased metabolic mechanisms enabling perceptual and active inference. The Stressed Body concept looks at the deleterious effects of unresolved uncertainty: in simple terms, somatic wear and tear or weathering in continual active inference in no-win situations.

The authors outline three aspects of stress: first, stress is evoked by situations with novelty, unpredictability, and uncontrollability; secondly, there must a sense of threat; and third, there must be multiple options for potentially uncertainty-resolving behavior. They then invoke three processes to resolve uncertainty: attention, learning, and habituation. In brief, information hungry Bayesian brains become hypervigilant to reduce uncertainty about strategy selection (information), and they selfishly need extra energy (thermodynamics) that is provided by the stress response.

Long-term stress, however, wears you out; you accumulate allostatic load. To alleviate uncertainty, goal-directed decision making is seen as active inference, that is, changing the probability distribution of relations of three types of states: 1, the current states of body or world; 2, attainable states (repertoire of actions/what you can do); and 3, goal states (where you would like to be). If you’re confident that your actions will work, then you produce motor output. If you’re uncertain, then you initiate an “emergency program” to get information to update beliefs and get inferences back in good shape. That is, stress reaction will sharpen senses/attention and increase learning via release of cortisol and catecholamines.

The upshot is that living through a no-win situation requires a juggling of multiple future scenarios, a cognitive load that has both affective and physiological consequences (see also The Guardian, 2020). The affective angle of irresolvable anxiety is matched by a physiological cost, per the “Selfish Brain” hypothesis, as fruitlessly searching to resolve uncertainty in choice of action in an essentially indeterminate future is itself a source of physiologically significant chronic stress. That is to say, in a no-win situation, if you don’t revise down your expectations in “adaptive preference” (crudely speaking, being resigned to your fate), if you keep fighting to find a solution, if you stay “resilient” in the face of change, as we are endlessly told to do, then you become trapped in a hypervigilant, cortisol-soaked state to consume and process as much information as possible.

But—and here is why the enactive interpretation of predictive processing the we will shortly turn to is important—the situation is ultimately political and material rather than epistemic; your problem is lack of power not lack of information, so continuing an information search to refine your risk probabilities doesn’t get you anywhere and in fact actually wears out your body, setting you up for an even worse case of Covid should you catch it.

## Enaction and chronic stress

The enactive approach to cognition (Varela et al., 1991; Thompson, 2007; Gallagher, 2017) sees cognition and action linked in brain-body-world coupled dynamic systems. For the enactivists, the boundaries of the cognitive agent extend not only beyond the brain, but beyond the skin; body, brain, and world are structurally coupled or mutually co-determining, to use the enactivist terminology. The overall enactive complaint is that for restricted or classically neurocentric processing models, the body is only there as a support (it provides energy to the brain or modulates attention and learning by glucocorticoids) and the world is only there a brake on perception as controlled hallucination. In other words, for neurocentrists, the brain is in central command mode, rather than being part of an overall system.

Kirchhoff and Kiverstein (2019) adopt Hurley (1998) notion of “extended dynamic singularity” or nexus of brain, body, world coupled dynamic systems. Kirchhoff and Kiverstein provide an account of the realization base of phenomenal consciousness extended by social and cultural practices; in Fanon’s critical phenomenological terms, this would be a matter of sociogenic lived experience: “what it is like” to be a black man cannot be divorced from its social setting, as being black in a world of anti-black racial colonialism is different from such experience in a differently racialized social setting.

Autonomous systems have self-production and self-distinction; enactive sense-making is historical (path-dependent) as experience locks in habits and extended to sometimes include active engagement with other agents that produce participatory sense-making in which coupled dynamics have a life of their own (Di Paolo et al., 2021). So intersubjectivity for enactivists is not just mind-reading, that is, inferring hidden mental states to predict action, but entering dynamics with other agents whose emotions affect you. For our purposes, the enactivist challenge amounts to insisting that some problems are political rather than (purely) epistemic. You act to change the world to improve the flourishing of those with whom you interact, not just to change sensory inputs to better fit predictions.

When it comes to consciously accessible pondering of options, it’s intuitively clear, I believe, that you can get worn out consciously “ruminating,” or endlessly going over options in a no-win situation. There is a physiological cost even to sub-personal, off-line simulations in conditional mode (“what would happen if I adopted this strategy?”). In high-stakes situations, with high-risk scenarios abounding on all sides, you get some allostatic load even before you act and get the real-world feedback that predictive processing is supposed to save you from (Peters et al., 2017).

People in vulnerable social positions, that is, deleterious affective frames, then, are doubly punished, especially in double-bind or no-win situations: even thinking about your options can exert a physiologically inscribed emotional cost, and then you must absorb the cost of the real-world feedback on top of that. In other words, in traditionally conceived allostatic action, you’re trying to change the world to restore a stable environment fit to habitual predictions. But if the world is recalcitrant, if the deck is stacked against you, then you get a double dose of allostatic load: not just a high cost to those allostatic actions which are swimming upstream in a racialized world, but there’s even a high cost to simple internal scenario generation.

## Case study: The death of Shenetta White-Ballard, an “essential worker”

Shenetta White-Ballard lost her life while serving as an “essential worker” in her job as a nurse in a senior care facility. Naming people as “essential worker,” and thus changing their attendant risk profiles, differs across political jurisdictions. In the case at hand, Louisiana had a fairly standard “Stay at Home” order (Office of the Governor of Louisiana, 2020), which included health care workers at the top of list of “Essential Worker Functions,” citing the Cybersecurity & Infrastructure Security Agency (CISA) guidelines of March 19 (Cybersecurity & Infrastructure Security Agency [CISA], 2020).

De Robertis (2020) (see also The Guardian, 2020) notes that Ms. White-Ballard suffered from a pre-existing condition: “A severe case of bronchitis followed by pneumonia had left the 44-year-old with chronic respiratory issues 2 years ago, so relying on oxygen to go about her daily life was a relatively new normal.” We don’t know the contribution of “weathering” here, but its presence here should be a plausible hypothesis.

As would be expected, White-Ballard’s employer reduces the situation to “choice” and willing assumption of risk on behalf of patients, thus naturalizing and individualizing the multiplicity or affective frame. DeRobertis reports, “Legacy representatives say White-Ballard made ‘a personal choice’ to continue working in an environment with COVID-19 positive patients.” Nothing is said about financial circumstances or forced choices. “Mrs. White, like many healthcare professionals across the country, chose to continue serving her resident population,” said Myles Holyfield, a Legacy spokesperson. “She did so with honor and professionalism. Shenetta is an example of risk that healthcare workers are willing to take while caring of the most vulnerable of our citizens.” A bit later in the article the mealy mouthed abnegation of corporate responsibility in the name of “choice” is made even more clear:

A Legacy representative said White-Ballard was aware of her options. Some employees chose not to work in the facility because of COVID-19 positive residents. Others took a short leave of absence and have since returned to work, while others have chosen to not return at all. “These choices are very personal to health care workers across the industry,” Holyfield said. “We, at Legacy, support those choices, whichever direction they may lead.”

The reporter follows up with a better, more concrete analysis than sheer “choice” of the affective frame that produced the entangled individuation of self-image as caring worker and financial and viral risk management facing White-Ballard: “Yet friends and family said she was terrified. As the member of her household with more secure financial footing, she felt a responsibility to keep working.” Here we see the affective term “terrified” linked to the status of the breadwinner responsible for the family; in the destruction of the social safety net, i.e., the Great Risk Shift, the family becomes the locus of private debt substituting for public policy, as we saw with Cooper. Another analysis from DeRobertis shows the double bind: “White-Ballard’s experience on the front lines of the coronavirus battle highlights the plight of many essential workers these past few months: continue to work at a job that places an employee at risk of infection, or walk away and face serious financial challenges.” Here we also see the predictive processing stress-load of constant risk calculation of viral and financial risk scenarios.

The conclusion of the article is gut-wrenching. First, we read of those left behind: “Eddie Ballard, Shenetta White-Ballard’s husband of 11 years, knows all too well the struggles of being ‘essential’ right now. He works at Walmart and has been given a 2-week period to mourn his wife’s death. His grief has debilitated him, he said, leaving him feeling aimless and unable to think clearly.” DeRobertis quotes Ballard:

“The only thing I’m doing right now is trying to hide it—I’m holding it down inside,” Ballard said. “I know I have to release it, but I can’t right now. I have too many things to do.” Now, he has to both raise and provide for their 14-year-old son alone. For this reason, Ballard said he “can’t completely break down” from his sadness. “I wish I could,” he said.

Turning again for the last time to White-Ballard’s situation, we see that self-subjectification as caring worker cannot ultimately be divorced from financial and viral risks. DeRobertis reports,

A coworker of White-Ballard, who did not wish to be named to protect her job, said she feels it is not a mystery why her colleague returned day after day to a place that put her in danger. “The same reason as to why she was willing to go to work is the reason a lot of people are still going to work,” she said. “They don’t have anyone else to do [the job]. It has to get done.”

At the very end of the article, DeRobertis re-establishes the double bind of affective workers who identify strongly with their caring for others: “Her husband said she never told him she dealt with COVID-19 patients; had he known, he would have demanded she find a way to stay home. Friends said she didn’t feel financially stable enough to walk away, but also that she loved her patients and cared for them deeply. ‘She had a beautiful heart,’ her husband said. ‘I want her to be known for her sacrifice.”’

## Conclusion

it’s a truism of critical social theory that there’s no such thing as a natural disaster. In Latourian terms, the SARS-CoV-2 virus is an actant in an Actor Network including social practices and individual human bodies as they have been shaped by social systems (Hanson, 2020). The very genesis of contemporary zoonotic diseases, to say nothing of their rapid spread, is connected to practices of financialization and globalization that collapse distances between wildlife and humans on the one hand and enable mass air travel on the other hand (Wallace et al., 2020). One could say that with COVID-19 we are in a period that is globalizing and intensifying the “domus” theorized by Scott (2017), Protevi (2019). Hence, to return to our Deleuzean framework, any one case of COVID-19 would be an individuation of the entire planetary disease system, including social, political, economic, and geographical aspects contributing to zoonotic origin, species transfer, propagation across nations, and then in a smaller scale, the multiplicity of transmission, including factors of ventilation, distance, time, activity, and immune systems.

We must remember that biopower did not begin in March 2020 with Covid “lockdowns.” Hence the desire to return to the “normal” rates for flu, asthma, COPD, and so on tempts us to overlook the multiple biopower dimensions that established the very same baseline rates to which we want to return. Furthermore, even keeping such a denaturalizing genealogy of the pre-Covid “normal” in mind as we scrutinize government Covid actions, we need to remember that not all “lockdowns” are the same, as different levels of government support are going to influence the fallout taking the form of increased rates of depression, anxiety, domestic violence, substance abuse, and so on.

As we have seen with Chowkwanyun and Reed, Covid illness factors are all subject to gender, race, class, and other analyses. A more fine-grained analysis would add to those factors status as “essential workers,” transport modes, intergenerational living, pre-existing conditions, and, as we will see, personal and familial financial risk-management. Following Adkins (2018) we have seen that underpaid essential health care workers faced increased virus risks to leverage their wages to take on the debt necessary to make ends meet. Many such people end up working for monthly payments to service their debt, which is no longer issued in view of repaying total, but of providing a securitizable revenue stream. So, we have a multi-dimensional regime of risk on viral, physiological, domestic, and institutional levels.

All these levels intersect governmental risk management in the naming of “essential workers” as those whose increased exposure to the Covid virus was an acceptable cost in implementing lockdowns: masks for all; remote work for some; in-person work for “essential workers.” A final dimension to the multiplicity that individuated in White-Ballard’s last days: as we noted above, emotional labor has a way of becoming a self-subjectifing practice, as many come to identify themselves with the caring aspects of a job. This only deepens the double bind: not only must you work for your family’s financial security, but you must also live up to your self-image as a caring health professional on the job, as well as providing emotional in additional to financial support to those at home.

In the reporting on White-Ballard’s death we see all the dimensions of this case study of Covid as an affective frame: distributed bio-social systems individuated as “bodies politic”; the neoliberal self-family-health-entrepreneur juggling risks in our epoch of biopower; the corporealization of racism in weathering as a subset of “political physiology”; and essential worker status forcing a complex juggling of financial and health risks in a no-win situation; and finally, an affective-cognitive “allostatic load” from that risk juggling that we can speculate exacerbated weathering-enabled pre-existing conditions and “co-morbidities” and contributed to the tragic death of Ms. White-Ballard.

## Data availability statement

Publicly available datasets were analyzed in this study. This data can be found here: https://www.theadvocate.com/baton_rouge/news/coronavirus/article_c2b7dd12-96e0-11ea-9ff8-9ff8faef31c2.html.

## Ethics statement

Ethical review and approval was not required for the study on human participants in accordance with the local legislation and institutional requirements. Written informed consent from people mentioned in the article was not required to participate in this study in accordance with the national legislation and the institutional requirements.

## Author contributions

The author confirms being the sole contributor of this work and has approved it for publication.
